# Chlorpromazine affects the numbers of Sox-2, Musashi1 and DCX-expressing cells in the rat brain subventricular zone

**DOI:** 10.1007/s43440-021-00259-7

**Published:** 2021-04-12

**Authors:** Jakub Skałbania, Artur Pałasz, Iwona Błaszczyk, Aleksandra Suszka-Świtek, Marek Krzystanek, Karina Paola Tulcanaz, John J. Worthington, Kinga Mordecka-Chamera

**Affiliations:** 1 Department of Histology, Faculty of Medical Sciences in Katowice, Medical University of Silesia, ul. Medyków 18, 40-752, Katowice, Poland; 2 Clinic of Psychiatric Rehabilitation, Department of Psychiatry and Psychotherapy, Faculty of Medical Sciences in Katowice, Medical University of Silesia, ul. Ziołowa 45/47, 40-635 Katowice, Poland; 3 Faculty of Medicine, Pontifical Catholic University of Ecuador, Av. 12 de Octubre 1076, 170143 Quito, Ecuador; 4 Division of Biomedical and Life Sciences, Faculty of Health and Medicine, Lancaster University, Lancaster, LA1 4YQ UK

**Keywords:** Adult neurogenesis, Chlorpromazine, Subventricular zone, Rats

## Abstract

**Background:**

Adult neurogenesis observed both in the subventricular zone (SVZ) and hippocampus may be regulated and modulated by several endogenous factors, xenobiotics and medications. Classical and atypical antipsychotic drugs are able to affect neuronal and glial cell proliferation in the rat brain. The main purpose of this structural study was to determine whether chronic chlorpromazine treatment affects adult neurogenesis in the canonical sites of the rat brain. At present, the clinical application of chlorpromazine is rather limited; however, it may still represent an important model in basic neuropharmacological and toxicological studies.

**Methods:**

The number of neural progenitors and immature neurons was enumerated using immunofluorescent detection of Sox2, Musashi1 and doublecortin (DCX) expression within SVZ.

**Results:**

Chlorpromazine has a depressive effect on the early phase of adult neurogenesis in the rat subventricular zone (SVZ), as the mean number of Sox-2 immunoexpressing cells decreased following treatment.

**Conclusion:**

Collectively, these results may suggest that long-term treatment with chlorpromazine may decrease neurogenic stem/progenitor cell formation in the rat SVZ and may affect rostral migratory stream formation.

**Supplementary Information:**

The online version contains supplementary material available at 10.1007/s43440-021-00259-7.

## Introduction

Clinical neuroimaging studies and several post mortem studies in patients with schizophrenia indicate a loss of neurons in the hippocampus, temporal cortex and prefrontal cortex, signposting cell apoptosis in these regions as part of the pathogenesis of schizophrenia [1]. For this reason, a significant concern is the potential effect of antipsychotic drugs (APD) on neurogenesis. If these drugs stimulate neurogenesis, their neuroprotective properties can potentially be used in schizophrenia pharmacotherapy. The majority of APDs that are applied to treat mental disorders with positive symptoms work through suppressing dopaminergic activity, although some newer drugs also affect serotonin receptors. Many studies point to the overactive dopaminergic system as a main cause of positive symptoms. This conclusion is supported by dopamine models of schizophrenia, drug action mechanisms, and research related to an attempt to induce certain elements of psychotic symptoms via dopamine precursors. The effect of APDs on neurogenesis has been repeatedly studied, initially it was thought that schizophrenia may have a destructive effect on neurons in the hippocampus and even inhibit neurogenesis [2], while all APD would reverse the damage and cause increased proliferation of nerve system cells. However, we now know that such a statement is an oversimplification, with recent studies demonstrating that there are some inaccuracies in the experimental data so far [3–5]. It is currently suggested that the drug category (typical/atypical) may also play an important role as these therapeutics have differing mechanisms of action [5]. Moreover, as reported by Respondek and Buszman [6], there are also discrepancies in the results of studies on dopaminergic modulation of adult neurogenesis in rats.

The synthesis of chlorpromazine in 1950, the first typical antipsychotic drug, was the breakthrough in psychiatric treatment of the time. This substance became the first medication that was able to distinctly improve the life quality of people suffering from schizophrenia. Although it opened a new era in psychopharmacology, in common with most first-generation drugs, it had several contraindications as well as serious side effects, such as extrapyramidal and Parkinson-like dysfunctions, weight gain, low blood pressure, depression and sleep disorder. For this reason, chlorpromazine is currently rarely used and it has been replaced by better-tolerated, more safe and effective second-generation drugs [7–10]. However, it remains a valuable model system for basic studies on dopaminergic signalling and pharmacology. Chlorpromazine crosses the blood–brain barrier and has high affinity to many receptors, explaining why this substance has numerous applications and a number of serious side effects. The action of the drug is based on antagonism—chlorpromazine blocks the following receptors [1, 7, 10]: dopaminergic: D_1_, D_2_, D_3_ and D_4_, serotoninergic: 5-HT_2_, 5-HT_6_, 5-HT_7_, histaminergic H_1_, adrenergic α_1_ and α_2_ receptors, M1 and M2 acetylcholine receptors. This effect is time-dependent and ultimately the reduced dopamine activity is responsible, among other things, for the antipsychotic properties of chlorpromazine.

Previous data on the effects of chlorpromazine on adult neurogenesis are limited, with few studies showing this APD has no effect on cell proliferation in the CNS, or that, like haloperidol, it inhibits neurogenesis [11]. There is also limited information indicating the neuroprotective properties of substances in epilepsy [12]. Moreover, it has recently been shown that the administration of a single dose of chlorpromazine reduces the number of DCX-positive cells in the rat hypothalamus, but that long-term treatment with this drug promotes the neuroblasts origin [13]. These conclusions are consistent with the fact that chlorpromazine initially increases dopaminergic activity which probably may adversely affect adult neurogenesis. The aim of the present experiment was to assess the effect of chronic chlorpromazine administration on specific stages of adult neurogenesis using immunohistochemical analysis of the expression of selected molecular markers of newborn neural cells (Sox-2, Musashi1 and DCX) in the brain of adult rats.

## Materials and methods

### Animals and drug administration

Studies were carried out on adult (2–3 months old, 180–220 g) male Sprague–Dawley rats from Medical University of Silesia Experimental Centre housed at 22 °C with a regular 12/12 light-darkness cycle with access to standard Murigan chow and water ad libitum. All procedures were approved by the Local Bioethic Committee at the Medical University of Silesia (decision no. 36/2012) and were conducted in a manner consistent with NIH Guidelines for Care and Use of Laboratory Animals. Two groups of animals (5 individuals each) received, respectively, control vehicle (saline) and chlorpromazine hydrochloride (*Fenactil,* pro injectione, 5 mg/ml, Polfa, Warszawa) at dose 10 mg/kg/day by intraperitoneal injection for 4 weeks. 24 h after the last administration, rats were quickly anaesthetized with isoflurane and then immediately sacrificed by decapitation.

### Immunofluorescence

**Fig. 1:**
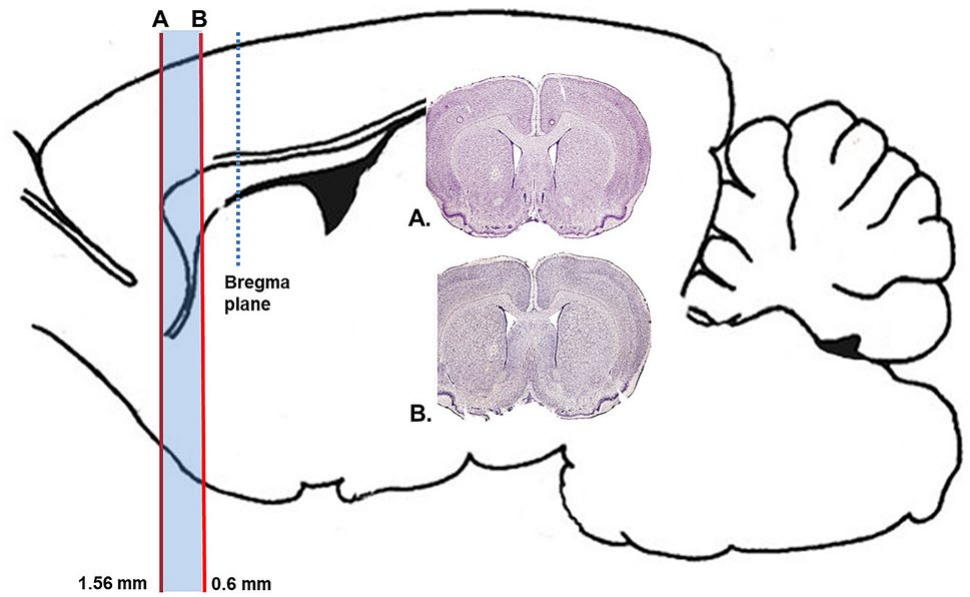
A scheme of sagittal view of the rat brain delineating planes at which tissue slices were made. The brain samples containing lateral ventricles with SVZ were coronally sectioned at the level 1.56 to 0.60 mm from bregma. Structural figures based on modified brain sections taken from the standard Paxinos and Watson The Rat Brain Atlas [14]

All brains were excised, fixed with 4% paraformaldehyde PBS (pH 7.2–7.4), dehydrated, embedded in paraffin and finally sectioned on the microtome (Leica Microsystems, Germany) in the coronal planes for subventricular zone SVZ; 1.56 to 0.60 mm from bregma, according to Paxinos and Watson [14] at 7 μm thick slices (Fig. 1.). After blocking with 5% goat serum, sections were incubated overnight with the following antibodies: monoclonal mouse anti-rat Sox-2 (1:1000, GeneTex; GTX627405), polyclonal rabbit anti-rat Musashi1 (1:200, GeneTex; GTX78273) and recombinant monoclonal rabbit anti-rat doublecortin (DCX) antibody [EPR19997] (1:200, Abcam; ab207175). Sections incubated with mouse/rabbit IgGs instead of primary antibodies were used as negative controls. After incubation with aforementioned primary antibodies, all brain sections were kept in darkness with secondary antibodies labeled with FITC or TRITC (1:200, Abcam) and then, mounted on slides with DAPI-containing medium. For calculation of DCX-positive cells, 10 slices (every tenth one) per rat for each brain region were used. All images (8 per slice) were captured with Nikon Eclipse *Ti* microscope (Nikon Instruments,Tokyo, Japan) and processed using Image ProPlus software (Media Cybernetics, USA). Anatomically comparable sections were analyzed and immunopositive cells were counted using ImageJ 1.43u software. To obtain density of immunostained cells per 100 µm of length, we counted the total number of Sox-2, Musashi-1 and DCX-positive cells in the neurogenic zones for each rat (which was the sum of cells from 10 slices) and divided the result per length of the analyzed subventricular regions (SVZ). Statistical analyses were performed using Statistica (Systat Software, San Jose, CA, USA). Differences between groups were statistically analyzed using non-parametric Mann–Whitney *U* test and they were considered significant at *p* < 0.05.

## Results and discussion

The present study reports that the long-term treatment with chlorpromazine, decreased the number of Sox-2 expressing cells in the rat brain SVZ. The results imply cautiously the effect of chlorpromazine on the early stages of adult neurogenesis, although its molecular mechanism is so far unexplained. In spite of the study limitations, such as low animal numbers and the lack of *Sox-2/Musashi-1* gene expression, our findings suggest that chronic treatment with chlorpromazine may depress adult neurogenesis in the rat SVZ and possibly impede the rostral migratory stream formation.

The effect of APDs on adult neurogenesis is heterogeneous in animal models, where data suggest that the greatest differences can be observed between typical and atypical APD; yet even within one group, there are some distinct internal differences [1, 5]. As stated previously, data on the impact of chlorpromazine on neurogenesis are limited, with few studies demonstrating contradicting results. Collectively suggesting that chlorpromazine may have various effects at different stages of neurogenesis and that the kinetics of prescribing may also be relevant. By analyzing the neurogenesis markers in the present study, we anticipated to reveal timing influences on the relationship of this antipsychotic drug with CNS stem cell proliferation.

**Fig. 2:**
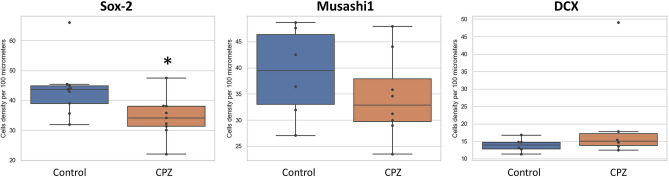
The number of Sox-2, Musashi-1 and DCX immunopositive cells in the subventricular zone of control rats and animals chronically treated with chlorpromazine (CPZ). Data are presented as median with ranges. Differences between groups were statistically analyzed using non-parametric Mann–Whitney *U* test and they were considered significant at *p* < 0.05

**Fig. 3:**
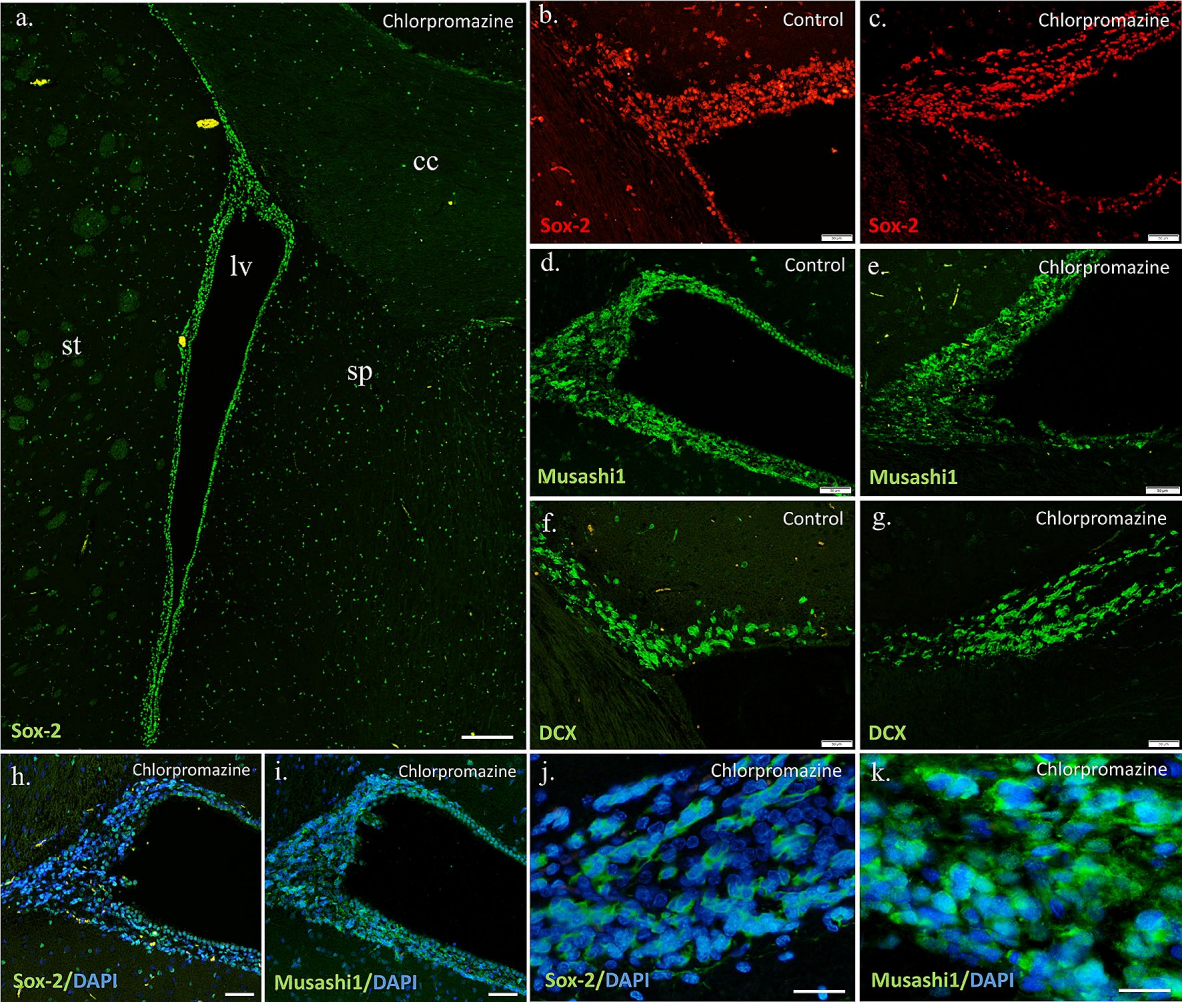
Representative expression of Sox-2, Musashi-1 and DCX in the rat SVZ cell populations. Images captured with Nikon Eclipse *Ti* microscope, magnifications used: x40, x200, x400. Scale bars: 200 µm (**a**), 50 µm (**b**–**g**, **j**), 100 µm (**h**, **i**), 20 µm (**k**). *cc* corpus callosum, *lv* lateral ventricle, *sp* septum, *st* striatum

According to some studies, the antagonistic affinity of the D_2_ receptor leads to an increase in neurogenesis. The fact that chlorpromazine among others blocks this receptor is an argument for the pro-neurogenic properties of this APD. Moreover, chlorpromazine does not block the D_5_ receptor, and stimulation of this receptor leads to an enzymatic cascade causing an increased tendency for cell proliferation [6, 15, 16]. Another important element of the pharmacodynamics of chlorpromazine in this context is blockade of the 5-HT_2_ receptor. Its blocking leads to the induction of the same enzymatic pathway as the stimulation of the D_5_ receptor [1, 17]. For the above reasons, it seemed reasonable to expect that chronic administration of chlorpromazine should support adult neurogenesis. Sox-2, (SRY [sex determining region Y]-box 2 is a transcription factor that is actively involved in the mechanisms of stem cell niche maintenance, their self-renewal and progenitor formation. However, as the cell grows, this factor decreases until silenced, and is therefore only an early stem cell marker. The Mann–Whitney *U* test revealed that treatment with chlorpromazine significantly decreased the number of Sox-2-immunopositive cells in SVZ compared to untreated controls (Figs. 2, 3; z = 2.119; N1 = 12; N2 = 10; *p* = 0.034) and the difference was 28%.

Another marker used in the study was the Musashi-1 (MSI1), RNA-binding protein with distinct expression in neural progenitor cell populations. Chlorpromazine administration decreased the number of Musashi1-immunopositive cells in SVZ compared to controls (Figs. 2, 3; *z* = 0.968; N1 = 0.12; N2 = 10; *p* = 0.332) and the difference was 19.97%; however, it was not statistically significant. Further research would dispel doubts as to whether the downward trend observed here is a real effect of the administered drug, or rather a coincidence resulting from the internal diversity of the studied animals. Nevertheless, the fact that Sox2-expressing cells are reduced under the influence of chlorpromazine, suggest that a similar phenomenon should be observed using the marker Musashi-1, as the expression of these factors is closely related to progenitor cells at a similar level of development.

The inverse trend was observed during examination of DCX-positive cells—a 7.89% increase in the number of cells was detected in the test group as compared to the control group. The Mann–Whitney *U* test revealed that treatment with chlorpromazine slightly increased the number of DCX-immunopositive cells in SVZ compared to untreated controls (Figs. 2, 3; *z* = 0.880; N1 = 12; N2 = 10; *p* = 0.378). However, this result is not statistically significant, which does not allow for final conclusion. Nonetheless, two possibilities can be assumed hypothetically. Either cells stained for DCX expression do not change their quantity after chronic administration of chlorpromazine, or there is some increase in cell density. In both cases, the same process occurs, but with a different intensity. As previously shown, cells reactive to the Sox2 antibody decreased in number as a result of drug administration, which makes it much less possible for later DCX-expressing cells to develop. Thus, it seems logical to hypothesize that the study group would have a lower density of DCX-immunopositive cells. However, for some reason, their number is equal or greater than the control group. This leads to the conclusion that despite the reduced potential (by reduced number), early progenitors and stem cells in chlorpromazine group had higher survivability compared to the control group (in which the development of nerve cells was undisturbed). Therefore, it can be hypothesized that chlorpromazine has a potential neuroprotective effect on maturing nerve cells (neuroblasts). For instance Kuruba et al. [12] mentioned the neuroprotective effect of chlorpromazine in CNS. In this study, no increase in the number of DCX-positive neuroblasts in SGZ was observed as a result of drug administration.

Overall, the effect of chlorpromazine on postnatal neurogenesis remains inconclusive. The present study showed that, in fact, chlorpromazine reduces the number of stem cells and early progenitors (Sox-2). Surprisingly however, this effect does not appear to affect the later stages of neuron formation. However, the upward trend in the number DCX-positive neuroblasts after drug treatment suggests cautiously that chlorpromazine may support the late stage of adult neurogenesis.The suppression of Sox-2+ progenitors origin by chlorpromazine may potentially be caused by the inhibition of dopamine receptors because neural stem cells (NSCs) in the rat SGZ express D2 receptors [6]. In contrast to chlorpromazine, clozapine, a second-generation antipsychotic (atypical) drug, was pro-neurogenic and anti-apoptotic. More than a two-and-a-half-time increase in the number of new cells in SGZ in the rat was demonstrated after clozapine treatment, but it did not affect the total number of new cells after three weeks. For this reason, the authors concluded that clozapine does not have protective properties against newly formed nerve cells [18]. An anti-apoptotic effect of clozapine was also observed [19]. Another atypical antipsychotic olanzapine, supports adult neurogenesis mainly within the SVZ, although there are some reports that also suggest this effect occurs in the SGZ [1]. It should be taken into account that the proliferative rates in the SVZ and in the SGZ are different. Moreover, an increased proliferation of progenitor cells in the rats’ prefrontal cortex was observed as a result of a 3-week treatment of this drug [20]. The results of the studies conducted so far show that haloperidol does not stimulate neurogenesis in the hippocampus [18] while it is stimulated by olanzapine [20], and some atypical neuroleptics reverse the inhibition of neurogenesis caused by repeated restraint stress (quetiapine) [21] and phencyclidine (clozapine, risperidone) [22, 23]. Based on these observations, a hypothesis has been formulated that it is atypical APDs, and not typical ones, that stimulate neurogenesis in the hippocampus. This hypothesis, however, is not confirmed, for example, by the studies of Halim et al. [20] who showed that clozapine does not stimulate neurogenesis in the hippocampus. The neuroprotective properties of olanzapine have also been proven and it is believed that the higher level of secretion of brain-derived neurotrophic factor (BDNF) and neural growth factor (NGF) is induced after treatment with olanzapine [6]. There is also a study reporting that adult neurogenesis in the hibernating hamsters (in the state of torpor) is more reduced in the SVZ than in the SGZ. Interestingly, this proliferative rate is normalized after 3–4 days of artificial hibernation. Of note, tau3R protein with microtubule-binding subunits turned out to be an appropriate marker for the analysis of canonical adult neurogenesis in this rodent species [24].

Data about another classic APD, haloperidol diverge, but most sources indicate that haloperidol does not increase the proliferation and vitality of newborn cells [3, 20, 21]. It has been suggested that this neuroleptic may exert pro-apoptotic effects on adult neurogenesis via inhibiting BDNF secretion [5]. However, there are also reports suggesting proneurogenic properties of haloperidol [4, 15]. It is possible that differences in experimental data are caused by use of different markers and their limited selectivity/affinity for specific neurogenic cells or even alterations in microbiota at differing sites of study that can influence APD function [15].

The mechanism responsible for the neurogenesis promoting effects of APD varies greatly due to the broad spectrum of the drugs themselves. Starting with typical APD that primarily affect dopamine D_2_ receptors by blocking them. Research shows, although not entirely clearly, that stimulation of the D_2_ receptor, which is located on the surface of CNS stem cells, inhibits proliferation. Thus, blocking the receptors by some APD may be one of the mechanisms enhancing neurogenesis (studies that have shown a positive effect of haloperidol on neurogenesis usually refer to this D_2_ receptor antagonistic mechanism of drug action) [6, 15, 16]. Stimulation of the D_5_ receptor, e.g. by ziprasidone—a second-generation antipsychotic agent, causes phosphorylation of Akt, which reduces the activity of the glycogen synthase kinase 3β (GSK-3β) enzymatic cascade. Inhibition of GSK-3β activates β-catenins and p53 and finally triggers cyclin D1 action, which in turn induces neurogenesis. Despite the research conducted so far, it is still unknown what element of the antipsychotic drug mechanism is responsible for stimulating neurogenesis [1]. Many reports show that the discussion dealing with the stimulatory effects of APD on adult neurogenesis requires detailed further study.

## Conclusion

The effect of chlorpromazine on postnatal neurogenesis in an animal model is still unclear. Despite the limitations of this study, including low number of rats and the lack of assessment of marker genes expression, we showed that chlorpromazine decreased the number of Sox-2-expressing stem cells and early neural progenitors in the rat subventricular neurogenic site.

## Supplementary Information

Below is the link to the electronic supplementary material.

Supplementary file1 (DOCX 13 kb)
