# Supplementary material for: Chlorpromazine affects the numbers of Sox-2, Musashi1 and DCX-expressing cells in the rat brain subventricular zone
Source: Pharmacol Rep. 2021 Apr 12;73(4):1164–9. doi: 10.1007/s43440-021-00259-7 (PMC8413197; doi:10.1007/s43440-021-00259-7)
Supplement: Supplementary file 1 — Supplementary file1 (DOCX 13 kb) [file 43440_2021_259_MOESM1_ESM.docx]

| Antibody | Host | Clonality | Reactivity | Isotype | Immunogen |
| --- | --- | --- | --- | --- | --- |
| **SOX2 antibody** clone [GT1352]  Cat. GTX627405 GeneTex | Mouse | Monoclonal | Rat, Mouse, Human Zebrafish, Sheep, Bovine, Cat, Chicken, Pig, | IgG2a | Recombinant protein encompassing a sequence within the center region of human SOX2. The exact sequence is proprietary |
| **Musashi 1** antibody  Cat. GTX78273  Gene Tex | Rabbit | Polyclonal | Human, Mouse, Rat | IgG | residues 5-21 [APQPGLASPDSPHDPCK] of the human, mouse and rat Musashi 1 protein |
| Recombinant Anti-**Doublecortin** antibody [EPR19997] | Rabbit | Monoclonal | Mouse, Rat, Human | IgG | Recombinant fragment. This information is proprietary to Abcam and/or its suppliers. |
